# Safety and efficacy of helical tomotherapy following lung-sparing surgery in locally advanced malignant pleural mesothelioma

**DOI:** 10.1007/s00066-023-02174-7

**Published:** 2023-11-22

**Authors:** Julian P. Layer, Pascal Fischer, Cas S. Dejonckheere, Gustavo R. Sarria, Rebekka Mispelbaum, Tessa Hattenhauer, Shari Wiegreffe, Andrea R. Glasmacher, Katharina Layer, Youness Nour, Lara Caglayan, Franziska Grau, Thomas Müdder, Mümtaz Köksal, Davide Scafa, Frank A. Giordano, Alberto Lopez-Pastorini, Erich Stoelben, Leonard Christopher Schmeel, Christina Leitzen

**Affiliations:** 1 Department of Radiation Oncology, University Hospital Bonn/Venusberg-Campus 1, 53127 Bonn, Germany; 2 Institute of Experimental Oncology, University Hospital Bonn, Bonn, Germany; 3 Department of Thoracic Surgery, St. Hildegardis Hospital, Augustinerinnen Krankenhäuser gGmbH, Cologne, Germany; 4 Department of Oncology, Hematology, Rheumatology and Immune-Oncology, University Hospital Bonn, Bonn, Germany; 5 Department of Radiation Oncology, University Medical Center Mannheim, Mannheim, Germany; 6 DKFZ-Hector Cancer Institute of the University Medical Center Mannheim, Mannheim, Germany

**Keywords:** Intensity-modulated radiotherapy, Local tumor control, Survival, Adjuvant radiotherapy, Trimodal therapy

## Abstract

**Purpose:**

To assess the value of radiation therapy (RT) with helical tomotherapy (HT) in the management of locally advanced malignant pleural mesothelioma (MPM) receiving no or lung-sparing surgery.

**Methods:**

Consecutive MPM cases not undergoing extrapleural pneumonectomy and receiving intensity-modulated (IM) HT were retrospectively evaluated for local control, distant control, progression-free survival (PFS), and overall survival (OS). Impact of age, systemic treatment, RT dose, and recurrence patterns was analyzed by univariate and multivariate analysis. As a secondary endpoint, reported toxicity was assessed.

**Results:**

A total of 34 localized MPM cases undergoing IMHT were identified, of which follow-up data were available for 31 patients. Grade 3 side effects were experienced by 26.7% of patients and there were no grade 4 or 5 events observed. Median PFS was 19 months. Median OS was 20 months and the rates for 1‑ and 2‑year OS were 86.2 and 41.4%, respectively. OS was significantly superior for patients receiving adjuvant chemotherapy (*p* = 0.008).

**Conclusion:**

IMHT of locally advanced MPM after lung-sparing surgery is safe and feasible, resulting in satisfactory local control and survival. Adjuvant chemotherapy significantly improves OS. Randomized clinical trials incorporating modern RT techniques as a component of trimodal treatment are warranted to establish an evidence-based standard of care pattern for locally advanced MPM.

## Introduction

Malignant pleural mesothelioma (MPM) is a rare and highly aggressive intrathoracic malignancy mainly associated with exposure to asbestos fibers. Prognosis is dismal despite multimodal treatment [1, 2]. While MPM derives from mesothelial pleural cells and infiltrates the lung first, diagnosis at a more extensive stage of disease is common, thus limiting survival dramatically [3] and rendering surgical care-only patterns detrimental. Palliative care options have improved lately, with targeted approaches [4] such as the addition of bevacizumab to chemotherapy [5] or dual checkpoint inhibition with ipilimumab and nivolumab [6]. However, defining the adequate multidisciplinary treatment patterns for operable MPM remains challenging, as critical patient assessment and selection are crucial and there is no general consensus on local treatment of MPM. Surgical options include extrapleural pneumonectomy (EPP) or extended pleurectomy/decortication (P/D) [7]. While only EPP as a radical surgery approach provides a truly curative intent [8], its associated high mortality and morbidity rates restrict the procedure clearly [9]. Further controversy persists on the role of neoadjuvant and adjuvant systemic therapy or adjuvant radiotherapy (RT) [10–12]. Previous reports demonstrated superior outcome for multimodal treatment approaches [13, 14]. Intensified RT may yield improved local control but was technically difficult in previous times due to usually large and irregularly shaped target volumes. This issue has been greatly alleviated by optimization of intensity-modulated RT (IMRT), enhancing organ at risk (OAR) preservation, particularly of the non-affected lung [1, 15]. We previously reported on the dosimetric feasibility of helical tomotherapy (HT) and volumetric modulated arc therapy (VMAT) in MPM [16, 17]. However, clinical data supporting the use of these RT techniques are still widely lacking. The combination of lung-sparing surgery and intensity-modulated (IM), image-guided (IG) HT may provide a rationale for an effective, quality of life-preserving therapy for patients deemed unfit for radical resection. Thus, we here sought to assess the outcomes of consecutive MPM patients undergoing RT using IG/IMHT.

## Methods

### Patients

The study collected data from consecutive MPM patients referred to the Department of Radiation Oncology at the University Hospital Bonn between January 2009 and September 2020 who had undergone HT following lung-sparing surgery (either biopsy only or P/D). In all enrolled cases, MPM was histopathologically confirmed. The data were retrospectively collected and filtered using Excel 2019 (Microsoft, Redmond, WA, USA). The collected information included, among other things, sociodemographic characteristics, date of primary diagnosis, primary tumor location, RT treatment characteristics, systemic treatment characteristics, histopathological tumor characteristics, recurrence patterns, and survival. All patients received follow-up examinations and imaging as per standard of care.

### Radiotherapy

For treatment planning, a computer tomography (CT) scan was acquired in supine position with elevated arms and 3 mm CT slice thickness. Clinical and planning target volumes were delineated according to Minatel et al. [13], covering the complete pleura in all cases. All patients received IG/IMRT with HT plans created using the Tomotherapy HiArt^®^ planning system (Accuray, Sunnyvale, CA, USA). We previously established dose constraints for OARs [16]. Prior to daily RT, a megavoltage computed tomography (MVCT) scan was obtained and matched to the planning CT to ensure accurate patient positioning. RT was carried out using a TomoTherapy^®^ (Accuray) linear accelerator with a single dose prescription of 1.8 to 2 Gy to a total dose of 45 to 60 Gy.

### Study endpoints

The primary endpoints of the study were local control (LC), distant control (DC), progression-free survival (PFS; defined as the date of RT initiation to the date of radiographically confirmed progression), and overall survival (OS; defined as the date of RT initiation to the date of death). Patients who were lost to follow-up were censored at the last timepoint of follow-up. In case of death prior to disease progression, the death date was censored and used as date of progression. The secondary endpoint of the study was RT toxicity according to the National Cancer Institute’s Common Terminology Criteria for Adverse Events (CTCAE) v5.0.

### Literature search

International literature databases (MEDLINE) and study registries (National Clinical Trials) were screened for similar retrospective and prospective reports on MPM treatment using the search terms “pleural mesothelioma” and “IMRT.” Available data were extracted and summarized.

### Statistical analysis

Excel 2019 (Microsoft) and GraphPad Prism (version 9, GraphPad Software, Boston, MA, USA) were used for data analysis. If not stated otherwise, statistical tests and analyses were performed as indicated in the respective figure legends. Figures were generated using GraphPad Prism 9 and Adobe Illustrator 2021 (Adobe Inc., San Jose, CA, USA). LC, DC, PFS, and OS were assessed by the Kaplan–Meier method and compared using the log-rank test. For multivariate analysis, the Cox proportional hazard model was used. Herein, the included variables were sex, age, neoadjuvant chemotherapy, adjuvant chemotherapy, surgery, total RT dose, and pattern of recurrence (in-field vs. distant). For all statistical tests, significance was defined as a *p*-value of less than 0.05.

## Results

### Patient characteristics

**Fig. 1:**
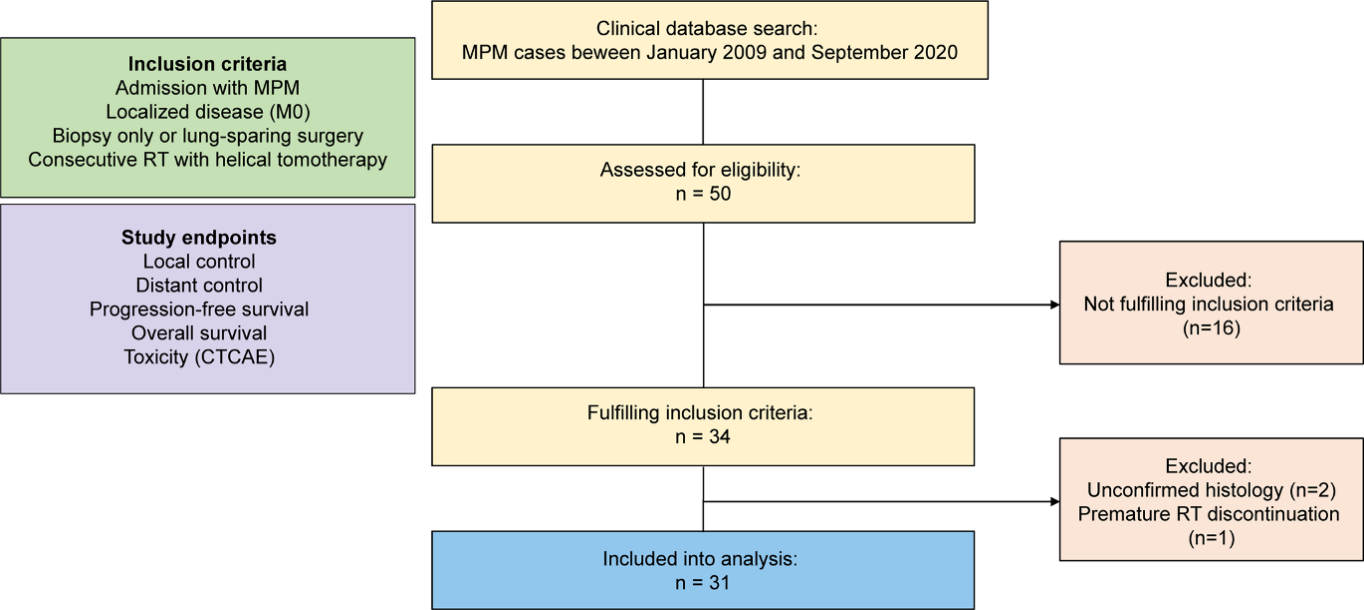
CONSORT diagram with inclusion criteria and study endpoints. *CTCAE* National Cancer Institute’s Common Terminology Criteria for Adverse Events, *MPM* malignant pleural mesothelioma, *RT* radiotherapy

Out of 50 cases admitted with MPM, a total of 34 localized MPM cases undergoing RT were identified. Of these, clinical follow-up data were available for 31 patients who had completed the RT (Fig. 1). Median patient age was 65 (range 46–77) years and 21 patients (67.7%) had undergone either total or partial surgical pleurectomy prior to RT. No patient had received EPP. Neoadjuvant platinum-based chemotherapy had been received by 18 patients (58.1%) and 13 (41.9%) have received adjuvant platinum-based chemotherapy. Two patients (6.5%) had not received any perioperative systemic therapy. Further patient characteristics are summarized in Table 1.

**Table 1:** Patient characteristics (*n* = 31)

Variable	*n* (%)	Median (range)
*Gender*		
Male	24 (77.4)	–
Female	7 (22.6)	
Age (years)	–	65 (46–77)
*Localization*		
Left	11 (35.5)	–
Right	20 (64.5)	
*Histology*		
Epitheloid	27 (87.1)	–
Biphasic	4 (12.9)	
*Chemotherapy*		
Neoadjuvant	18 (58.1)	–
Adjuvant	13 (41.9)	
None	2 (6.5)	
*Surgery*		
Total pleurectomy	18 (58.1)	–
Partial pleurectomy	3 (9.7)	
Biopsy only	10 (32.3)	
*RT technique*		
Helical tomotherapy	31 (100)	–
RT dose (Gy)	–	50.4 (45–60)
Follow-up (months)		20 (5–96)

*RT* radiotherapy

### Toxicity

In general, RT was well tolerated, and the majority of adverse events were mild. The most common events of all grades were fatigue (60.0%), dysphagia (50.1%), and nausea (40.0%). A grade 3 event was experienced by 26.7% of patients (dysphagia 16.7%, dyspnea 6.7%, pain 3.3%). There were no grade 4 or 5 events observed. One patient discontinued treatment due to an unrelated endocarditis and was thus not considered for the outcome analysis. A complete listing of the adverse events can be found in Table 2.

**Table 2:** Adverse events

Event	*n* (%)
*Fatigue*	
Grade 1	9 (30.0)
Grade 2	9 (30.0)
*Nausea*	
Grade 1	4 (13.3)
Grade 2	8 (26.7)
*Pain*	
Grade 1	5 (16.7)
Grade 2	4 (13.3)
Grade 3	1 (3.3)
*Radiodermatitis*	
Grade 1	7 (23.3)
Grade 2	5 (16.7)
*Dysphagia*	
Grade 1	5 (16.7)
Grade 2	5 (16.7)
Grade 3	5 (16.7)
*Dysgeusia*	
Grade 1	2 (6.7)
*Cardiac*	
Grade 1	2 (6.7)
*Dyspnea*	
Grade 1	3 (10.0)
Grade 2	6 (20.0)
Grade 3	2 (6.7)
*Cough*	
Grade 1	5 (16.7)
Grade 2	1 (3.3)

### Outcome

**Fig. 2:**
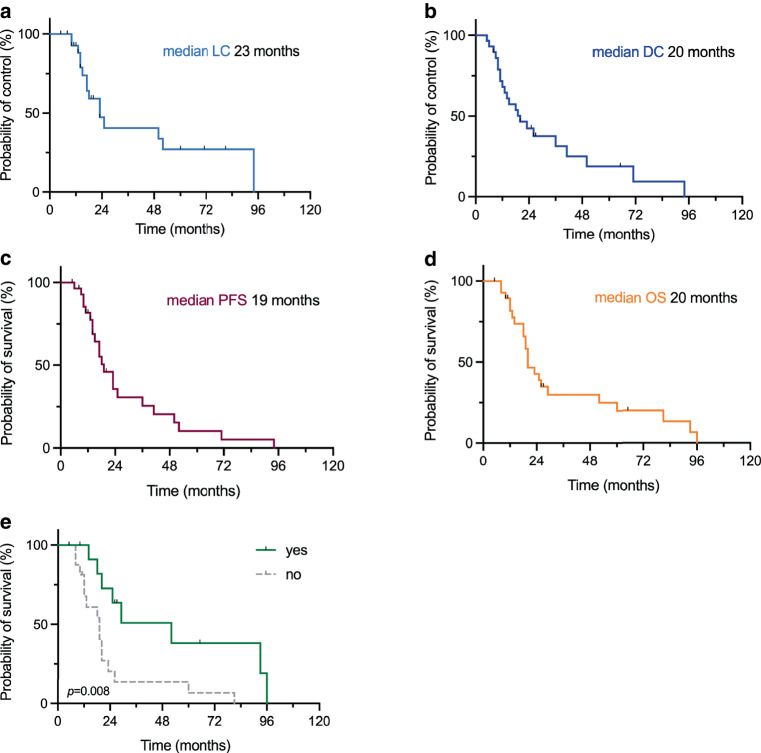
Outcome analysis. Kaplan–Meier curves for local control (**a**), distant control (**b**), progression-free survival (**c**), overall survival (**d**). **e** Overall survival in dependency on adjuvant chemotherapy, Kaplan–Meier curve with log-rank test. *DC* distant control, *LC* local control, *OS* overall survival, *PFS* progression-free survival

With a median follow-up of 20 (5–96) months, LC was maintained for a median of 23 months with 92.6% LC after 1 year, 47.3% after 2 years, and 40.6% after 3 years (Fig. 2a). The median DC time was 23 months and the DC rate was 69.0%, 37.9%, and 20.7% after 1, 2, and 3 years, respectively (Fig. 2b). The median PFS was 19 months (Fig. 2c). The 1‑year PFS rate was 72.4% and the 2‑year PFS rate 24.1%. The median OS was 20 months and the rates for 1‑year and 2‑year OS were 86.2 and 41.4%, respectively (Fig. 2d). In a multivariate analysis, there were no factors significantly associated with improved PFS. The same analysis for OS revealed a significant risk reduction for adjuvant chemotherapy (*p* = 0.001), which was confirmed in univariate analysis (Fig. 2e).

## Discussion

MPM remains a hard-to-treat tumor entity lacking evidence-based, standardized guidelines on therapeutic management in the locally advanced setting. We here retrospectively assessed the safety and outcome of MPM treatment with IG/IMHT in a single-center approach, reporting tolerable toxicity as well as promising LC and OS, wherein additional adjuvant chemotherapy was associated with improved outcome.

**Fig. 3:**
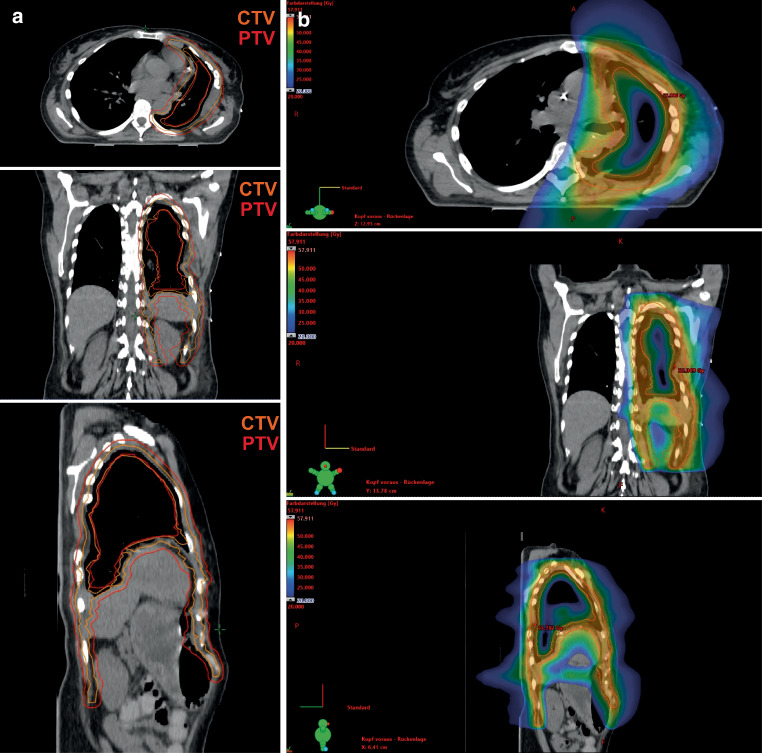
Exemplary illustration of target volume delineation (**a**) and dose distribution (**b**) with helical tomotherapy in a 60-year-old female with epitheloid malignant pleural mesothelioma receiving 1.8 Gy to 50.4 Gy following neoadjuvant chemotherapy (cisplatin and pemetrexed) and extended pleurectomy/decortication. *CTV* clinical target volume, *PTV* planning target volume

RT is an established and safe procedure for palliative management of pain or obstruction in localized MPM [18, 19]. However, adequate dose delivery to the target volume in curative intent has been a major challenge for radiation oncologists for a long time and indications for RT must be well defined, taking into account treatment-related side effects. A randomized controlled phase III trial previously assessed routine irradiation of the surgical procedure tract, which was not included in the target volume in this collective, observing no significant benefit regarding local procedure tract metastases but also quality of life at the expense of increased early postinterventional toxicity [20]. The introduction of IMRT defined a new era, as it allows safe and effective curative-intent dose delivery to the MPM while sparing OARs sufficiently [15]. Adjuvant RT after P/D is particularly challenging as the lungs remain in situ and, thus, sparing of these OARs complicates dose delivery to the target volume. Ironically, this renders extensive and complicated surgery with radical EPP the easier approach for radiation oncologists. We have previously demonstrated the feasibility of adjuvant curative-intent RT after P/D with HT-based planning, thus improving potential lung and kidney sparing [17]. Although both HT (Fig. 3) and VMAT may provide very satisfying and comparable dose distributions, there are distinct characteristics and advantages for both treatment techniques. In our previous series, HT achieved optimal contralateral lung sparing, which may be considered the priority planning objective due to the relevant risks of impairing quality of life and potentially lethal events following pneumonitis. We also reported slightly superior target volume coverage for HT, but its clinical significance remains unclear. On the other hand, HT usually necessitates significantly longer beam-on time. Ultimately, the RT technique of choice requires consideration of individual patient-centered factors like clinical performance and compliance to patient positioning or breath-holding, and physical factors like the geometry of the designated target volume and adjacent anatomy.

While systemic chemotherapy is an established approach to improve both PFS and OS for MPM not eligible for curative surgery [21], the true prognostic role of both chemotherapy and RT in localized MPM remain undisclosed [22, 23]. This is mainly due to low overall patient numbers, but also a high clinical heterogeneity within the MPM patient collectives, which hampers patient recruitment for randomized trials. As such, even though IMRT has already become standard of care in developed countries [24], there are only limited data on its effect on RT outcomes in MPM thus far. Most available data focus on post EPP-RT. EPP is the most radical surgical option for localized MPM, which requires careful patient selection and is associated with severe toxicity [9]. Patients undergoing this extensive surgery may suffer from impaired mobility and overall reduced quality of life. While P/D was formerly considered a purely palliative procedure, recently, various studies have established a curative role for the procedure [7], making it the primary option for sensitive multimodal treatment building up on the advances in RT technology allowing for more precise, dose-escalated RT. In fact, our results are in line with previous reports on IMRT of MPM after EPP (Table 3), although all our patients received less extensive, lung-sparing surgery or no surgical resection at all. Besides the mentioned primary patient-centered benefits with reduced risk of mortality, toxicity, and in-hospital times, our results also suggest a treatment option which spares the limited economic resources and capacities of the health care system [25].

**Table 3:** Previous series on IMRT of localized MPM with relevant patient numbers

Publication	*n*	Surgery	RT technique	RT dose	HT (in %)	CTX (in %)	Tox	mPFS (months)	mOS (months)
Rosenzweig et al., 2012 [36]	16	Biopsy	IMRT	46.8 Gy^b^	0	89	44% G3+ 12.5%G4 12.5%G5	NA	17
	20	P/D	IMRT	46.8 Gy^b^	0	89		NA	26
Thieke et al., 2015 [2]	62	EPP (100%)	IMRT	52.6 Gy^b^	33	100	0% G4+	NA	20.4
Harrabi et al., 2017 [28]	10	P/D (100%)	IMRT	52.2 Gy^a^	100	100	0% G3+	13	19
Simon et al., 2018 [30]	26	EPP (58%)	IMRT	54 Gy^a^	0	67	0% G3+	26.7	34.9
Trovo et al., 2021 [14]	54	EPP (100%)	IMRT	50–60 Gy	31	70	31% G3+ 5.6% G5	NA	21
Arrieta et al., 2020 [37]	15	P/D (100%)	IMRT	48.7 Gy^a^	0	100	46.6% G3+	18.9	23.6
Nakanishi-Imai et al., 2022 [38]	25	Biopsy (20%) EPP (80%)	IMRT	50.4 Gy^a^	100	72	NA	NA	26
*Current series*	31	Biopsy (32%)	IMRT	50.4 Gy^a^	100	93.5	27% G3+	19	20
		P/D (68%)							

*CTX* chemotherapy, *EPP* extrapleural pneumonectomy, *HT* helical tomotherapy, *IMRT* intensity-modulated radiotherapy, *mOS* median overall survival, *mPFS* median progression-free survival, *P/D* extended pleurectomy/decortication, *RT* radiotherapy, *Tox* toxicity

^a^median dose, ^b^mean dose.

With the ongoing implementation of next-generation O‑ring linear accelerators into clinical practice, interest has increased in experiences with last-generation HT [26]. First reports on palliative HT of MPM suggest good efficacy at low toxicity [27]. Only a very small series of 10 patients [28] has previously assessed the role of IMRT HT for P/D patients, reporting an excellent toxicity profile. In the largest retrospective EPP series to date, Thieke et al. reported a 1-year OS of 63% [2], which was exceeded by far in this series with 86%. However, 2‑year OS was similar, with 42% each. Of note, we observed a very high LC rate of 92.6% after 1 year, which may have contributed to the improved overall outcome. The convincing DC rate of this series is most likely due to the systemic chemotherapy, as adjuvant chemotherapy was associated with a significant OS improvement. The described outcome benefit of trimodal MPM treatment is consistent with other reports [11, 29].

Given the acceptable toxicity profile of IMRT reported here but also elsewhere [2, 14, 30, 31], it remains debatable whether MPM patients might furthermore benefit from applying a sequential boost [29, 32]. In comparison to older studies incorporating outdated RT techniques with fatal events [33], the reported toxicity profile clearly demonstrates the benefit of modern RT techniques, as no grade 4 or 5 events were recorded. Nevertheless, the grade 3 toxicity rate of more than 25% presented here has to be taken into account in patient counseling and clinical decision-making, as meticulous patient selection for this intensive treatment regimen remains crucial. Additionally, as yet widely unestablished standardized patient monitoring and early management of occurring side effects may alleviate symptom burden and prevent high-grade toxicity [34, 35].

Our study has several limitations. The small sample size did not allow for a more detailed analysis of prognostic factors or stratification such as patients receiving trimodal treatment versus RT or chemotherapy only. Equally, the long interval between the first and last patients included may cause a bias due to the heterogeneity in care patterns. However, MPM is a rare disease and clinical outcome reports of RT are scarce. To the best of our knowledge, this is one of the largest cohorts reported thus far accounting for technical progress that has been made recently. More importantly, this is the first report on the safety and outcome of IG/IMHT of MPM with a relevant patient number.

Due to the lack of randomized clinical trials, the debate on the optimal treatment pattern for locally advanced MPM is likely to continue. A single-center phase II trial recently reported convincing long-term outcomes for neoadjuvant IMRT followed by EPP at the costs of high toxicity [39]. An ongoing phase III randomized clinical trial may soon shed some light on the optimal treatment pattern for locally advanced MPM [40].

## Conclusion

HT of MPM after lung-sparing surgery is safe and tolerable and results in satisfactory local control and overall survival. Adjuvant chemotherapy can furthermore improve the clinical outcome. However, careful patient selection for trimodal treatment remains crucial to avoid imbalanced toxicity. Randomized clinical trials are warranted to confirm these findings and to establish trimodal treatment as an evidence-based standard of care pattern for locally advanced MPM.
